# Evaluation of the prevalence of *Aeromonas* spp., *Campylobacter* spp., and *Clostridioides difficile* in immunocompromised children with diarrhea

**DOI:** 10.1186/s12879-024-09372-3

**Published:** 2024-05-22

**Authors:** Hosein Heydari, Abolfazl Iranikhah, Ahmad Ghasemi, Abolfazl Mohammadbeigi, Seyed Ali Sadat-Mirei, Saeed Shams, Somayeh Kermani

**Affiliations:** 1 Pediatric Medicine Research Center, Qom University of Medical Sciences, Qom, Iran; 2 Gastroenterology and Hepatology Diseases Research Center, Qom University of Medical Sciences, Qom, Iran; 3 Department of Microbiology, Research Center of Reference Health Laboratories, Ministry of Health and Medical Education, Tehran, Iran; 4 Department of Epidemiology and Biostatistics, School of Health, Qom University of Medical Sciences, Qom, Iran; 5 Cellular and Molecular Research Center, Qom University of Medical Sciences, Qom, Iran

**Keywords:** Immunocompromised, Children, *Aeromonas*, *Campylobacter*, *Clostridioides difficile*, Diarrhea, Co-infection

## Abstract

**Aim:**

Diarrhea is a common disease in immunocompromised patients and can be associated with greater morbidity and even mortality. Therefore, the present study was designed to determine the prevalence of *Aeromonas* spp., *Campylobacter* spp., and *C. difficile* among immunocompromised children.

**Methods:**

This study was conducted on 130 stool samples from patients with diarrhea who had defects in the immune system and were referred to Hazrat Masoumeh Children’s Hospital in Qom. Demographic information, clinical symptoms, immune status, and duration of chemotherapy were also recorded for each child. DNAs were extracted from the stool, and then direct PCR assays were done by specific primers for the detection of *Aeromonas* spp., *Campylobacter* spp., and toxigenic *C. difficile*, including *tcdA/B* and *cdtA/B* genes. Co-infection in patients was also evaluated.

**Results:**

60.8% and 39.2% were male and female, respectively, with a m ± SD age of 56.72 ± 40.49 months. Most cases of immunocompromised states were related to Acute Lymphocytic Leukemia (77.7%) and Non-Hodgkin Lymphoma (14.6%). 93.1% of patients were undergoing chemotherapy during the study. Among patients, most clinical symptoms were related to bloody diarrhea (98.5%) and fever (92.3%). Based on PCR, 14.6, 9.2, and 1.5% were positive for *Aeromonas* spp., *C. difficile*, and *C. jejuni*, respectively. Among the *C. difficile*-positive cases, the *tcdA* gene was only detected in one patient. In total, three co-infections were identified, which included *Aeromonas* spp./*C. difficile* (*tcdA*^+^), *C. jejuni*/*C. difficile*, and *C. jejuni*/*Aeromonas* spp.

**Conclusions:**

This is the first study in Iran to investigate the simultaneous prevalence of some pathogens in immunocompromised children with diarrhea. Because *Aeromonas* spp., *Campylobacter* spp., and *C. difficile* are not routinely detected in some laboratories, infections caused by them are underappreciated in the clinic. Our results showed that these pathogens are present in our region and can cause gastroenteritis in children, especially those with underlying diseases. Therefore, increasing the level of hygiene in some areas and controlling bacterial diarrheal diseases should be given more attention by health officials.

## Introduction

Infectious diarrhea is one of the most common gastroenterological diseases and a major problem worldwide. It is one of the most important causes of death, especially in children, in developed and developing countries [1–4]. In addition, patients with underlying conditions, such as an immunocompromised state, are at a higher risk for severe gastroenteritis infections caused by bacteria, viruses, fungi, and parasites, and the incidence of symptoms and lethality is higher than that of healthy people [5–7]. The most important bacteria causing diarrhea include *Campylobacter* spp., *Salmonella* spp., *Shigella* spp., *Yersinia enterocolitica*, *Vibrio cholerae*, *Aeromonas* spp., *Clostridioides difficile*, and intestinal pathotypes of *Escherichia coli* [8–10].

The genus *Aeromonas* (*A.*), with 36 species, belongs to the Aeromonadaceae family. These are rod-shaped, Gram-negative, and facultative anaerobic bacteria. This genus consists of mesophilic and psychrophilic bacteria that can grow in a wide range of temperatures from 10 to 42 °C. As environmental opportunists, these bacteria cause disease in a wide range of hosts, including humans, aquatic and terrestrial animals [11]. *A. hydrophila*, *A. caviae*, *A. veronii* biovar *sobria*, *A. dhakensis*, and *A. trota* are the most important species isolated from human feces [12]. In addition, these species can cause bacteremia, cellulitis, urinary tract infection, wound infection, and gastroenteritis in immunocompromised hosts. Gastrointestinal manifestations caused by these bacteria vary from mild diarrhea to cholera-like watery diarrhea and *Shigella*-like dysentery [13, 14]. Intestinal infection caused by these bacteria is mainly seen in children, adolescents, and people with underlying states, affecting 2–13% of children, 2–7% of adults with healthy immunity, and up to 13% of patients with immunocompromised states [15].

*Clostridioides difficile* (*C. difficile*), belongs to the Clostridiaceae family, is a Gram-positive, obligate anaerobic, rod-shaped, and spore-forming enteric bacterium. Strains producing toxins A (enterotoxin) and B (cytotoxin) are known as hospital-acquired pathogens and the causative agent of colitis and antibiotic-associated diarrhea. The symptoms and severity of the disease vary from simple colonization to the formation of the pseudomembranes on the colonic mucosal surfaces, sepsis, shock, and death [1, 16]. According to reports, immunocompromised people are more susceptible to *C. difficile* infections (CDIs) and show more severe clinical manifestations of this disease. In recent years, CDI cases have been increasing in hospitalized cancer patients, especially children, and have reached 25%. Many factors such as continuous contact with broad-spectrum antibiotics, inherent antimicrobial activity of some chemotherapy regimens, high contact with medical care devices, and the inhibitory effect of chemotherapy on the immune system increase the risk of CDIs in children [10, 17].

The genus *Campylobacter* (*C.*), belongs to the Campylobacteraceae family, comprises Gram-negative, motile, curved or spiral bacteria and can be seen in V or S shapes. These account for cases of gastroenteritis in developed and developing countries and causes a disease called campylobacteriosis. The most common species causing infection in humans are *C. jejuni* and *C. coli* with prevalence rates of about 90 and 10%, respectively [18–20]. This disease is a zoonotic disease and the main routes of its transmission to humans is through the consumption of contaminated water, vegetables, and food (including beef, pork, birds, and unpasteurized milk) [21]. The incubation period of the disease is 2–5 days and gastroenterological symptoms include diarrhea (which may be bloody), headache, nausea, vomiting, cramp, abdominal pain, cholecystitis, and fever [22]. Although *Campylobacter* infection is mostly non-fatal, there have been reports of patient deaths. For example, about 124 deaths caused by this infection are reported annually in the United States, and this rate is higher in developing countries [23]. Statistics also show that the prevalence of infection is high (even higher than shigellosis), especially in children under 5 years of age in developed and developing countries [24]. Prolonged infections and extra-intestinal manifestations caused by these bacteria have also been reported in patients with underlying conditions [25, 26].

Therefore, our aim was to detect the simultaneous presence of *Aeromonas* spp., *C. jejuni*, *C. coli*, and *C. difficile* in immunocompromised children with diarrhea, referred to a specialized children’s hospital using direct PCR, which was done for the first time in Iran.

## Methods

### Patients and sampling

This research was conducted on 130 immunocompromised patients with infectious gastrointestinal symptoms referred to Hazrat Masoumeh Children’s Hospital in Qom between 2018 and 2019. Sample size calculation was calculated considering the results of the Boustanshenas et al. study [27], which estimated the prevalence of *Aeromonas* spp. at 2.3%, confidence interval 95%, and error 0.03. Therefore, the minimum sample size was estimated to be 98 patients, and to increase the precision, 130 patients we enrolled in this study. Our inclusion criteria included consent, underlying disease, symptoms of infectious diarrhea, and no antibiotic use during diarrhea. Briefly, after obtaining written informed consent from the patients or their parents/guardians, 1 ml of fecal sample from each child was collected in a sterile container and then sent to the Cellular and Molecular Research Center of the university and stored at -20 °C until testing. In a questionnaire, demographic information, type of underlying disease, history of chemotherapy, and gastrointestinal symptoms for each patient including age, gender, travel, contact with animals, swimming, duration of diarrhea (days), bowel movements/day, fever, blood in feces, vomiting, cramp, and abdominal pain as well as some laboratory findings, were recorded.

### DNA extraction and polymerase chain reaction (PCR) for direct diagnosis of infections

From the stool samples, DNA was extracted by a modified method provided by Yang et al. [28]. Briefly, 200 µl of stool was centrifuged at 10,000 g for 5 min. The obtained supernatant was transferred to a new microtube and then centrifuged again at 13,000 g for 10 min. Next, in order to remove the PCR inhibitors, the precipitate was washed three times with acetone (each time, centrifugation was performed at 13,000 g for 10 min). In the last step, the supernatant was discarded, and 200 µl of sterile distilled water was added to the pellet. Genome extractions were done using the boiling method and stored at -20 °C until PCR.

Molecular identification of *C. jejuni* and *C. coli* was performed by the duplex-PCR method described in our previous study [29] for the *cadF* gene, while *C. difficile* and *Aeromonas* spp. were detected by separate PCRs based on other articles using *cdd3* and 16 S rRNA genes, respectively. The final volume of each reaction was 25 µL, including 10 µL of 1X Master mix (Ampliqon, Denmark), 1 µL of primers (10 pmol/µL) (Table 1), 3 µL of extracted DNA, and 10 µL of deionized water.

The amplification process consisted of an initial denaturation at 95 °C for 3 min (1 cycle), followed by 33 cycles including denaturation at 95 °C for 30 s, annealing temperature (Table 1), and elongation at 72 °C for 30 s. The final elongation step was performed at 72 °C for 5 min (1 cycle) (Thermocycler; Eppendorf, Hamburg, Germany). *Aeromonas hydrophila* ATCC 7966, *C. difficile* VPI10463, *C. coli* ATCC 43,478, and *C. jejuni* ATCC 29,428 were used as positive controls in the PCRs. The isolates that were positive for the *cdd3* gene in PCR were re-evaluated for the presence of toxigenic genes *tcdA*, *tcdB*, *cdtA*, and *cdtB*. The PCR products were loaded on a 1% agarose gel and evaluated by a gel documentation device with UV light (Thermo Fisher Scientific, USA).

**Table 1:** Primers used in this study

Bacteria	Target gene	Sequence ( 5’ → 3’ )*	Size (bp)	Annealing temperature (°C)/time(s)	Reference
*Aeromonas* spp.	16 S rRNA	F: GGGAGTGCCTTCGGGAATCAGA	356	55/45	[30]
		R: TCACCGCAACATTCTGATTTG			
*C. coli* *C. jejuni*	*cadF*	FU: TTGAAGGTAATTTAGATATG	461 737	43/30	[29]
		R1: TTTATTAACTACTTCTTTTG			
		R2: ATATTTTTCAAGTTCATTAG			
*C. difficile*	*cdd3*	F: TCCAATATAATAAATTAGCATTCCA	622	52/60	[31]
		R: GGCTATTACACGTAATCCAGATA			
	*tcdA*	F: GCATGATAAGGCAACTTCAGTGGTA	625		[32]
		R: AGTTCCTCCTGCTCCATCAAATG			
	*tcdB*	F: GGAAAAGAGAATGGTTTTATTAA	162		[33]
		R: ATCTTTAGTTATAACTTTGACATCTTT			
	*cdtA*	F: GGGAAGCACTATATTAAAGCAGAAGC	221		[34]
		R: CTGGGTTAGGATTATTTACTGGACCA			
	*cdtB*	F: TTGACCCAAAGTTGATGTCTGATTG	262		
		R: CGGATCTCTTGCTTCAGTCTTTATAG			

* All the items that were necessary for the evaluation of the primers, including the primer secondary structures, e.g. self- and cross-dimers, were re-examined using bioinformatics software.

### Statistical analysis

Results were reported using descriptive statistics including mean ± standard deviation (m ± SD) as well as frequency and frequency percentage. The frequency distribution of clinical symptoms and laboratory findings as well as the rate of infection and their comparison with other qualitative demographic variables, including the gender of the patients, were done using Fisher’s Exact and Chi-Square Tests. Independent t-test was also used to compare quantitative variables such as age in two groups of subjects. Statistical analysis was performed in SPSS 22 software (IBM, NY, USA) at a significance level of less than 0.05.

## Results

The m ± SD age of the patients was 56.72 ± 40.49 months, and 60.8% of whom were male (m ± SD age = 58.58 ± 43.99 months). Among them, 101 (77.7%), 19 (14.6%), and 10 (7.7%) had acute lymphocytic leukemia (ALL), non- Hodgkin lymphoma (NHL), and retinoblastoma cancer, respectively. 121 patients (93.1%) were undergoing chemotherapy during the study (with the m ± SD of 15.04 ± 11.95 months). In terms of clinical symptoms recorded in the questionnaire, 128 (98.5%), 120 (92.3%), 115 (88.5%), 98 (75.4%), 80 (61.5%), and 55 (42.3%) patients had bloody diarrhea, fever, anorexia, vomiting, weight loss, and cramp, respectively. The m ± SD of the number of days of diarrhea in our patients was determined as 2.93 ± 1.40 with a range of 1–9 bowel movements per day. None of the patients and their parents had detailed information about the consumption of contaminated food and water, the contact with animals, and travel related to the disease. According to the laboratory report of our hospital, the patient’s stool cultures were negative for *Shigella* spp., *Salmonella* spp., and *Escherichia coli* O157:H7.

Among the patients, 19 (14.6%) cases were diagnosed as positive for infection with *Aeromonas* spp., of which 57.9% were male and 42.1% were female (with a m ± SD age of 55.31 ± 46.35 months). There was no significant relationship between age and gender with *Aeromonas* infection (*P* > 0.05). The duration of diarrhea among *Aeromonas*-positive patients showed 2.73 ± 1.28 days (mean of 5.05 bowel movements/day). 94.7% of them had bloody diarrhea and 3.5% had watery diarrhea. In addition, 94.7, 84.2, 68.4, 52.6, 42.1, and 78.9% of *Aeromonas*-infected patients had anorexia, fever, weight loss, cramp, abdominal pain, and vomiting, respectively, but no significant relationship between the presence of infection and clinical findings. The presence of *Aeromonas* infection in patients with ALL accounted for the most cases (73.7%), followed by NHL with 21.0%. According to the season, the highest positive rate was related to autumn with 13 cases (68.0%), followed by winter with 4 cases (21.0%), and summer with 2 cases (11.0%). No significant difference between the number of positive cases of *Aeromonas* spp. with the autumn season was obtained (*P* = 0.631).

Of the 130 children, 12 cases (9.2%) were positive for *C. difficile* with a m ± SD age of 75.0 ± 52.51 months. 58.3% of them were female, and 41.7% were male. Among them, one case was infected with a toxin-producing strain (*tcdA*^+^) and no case of the presence of double genes (*tcdA/B* and *cdtA/B*) was observed. The duration of diarrhea in patients was recorded as 3.83 ± 2.28 days (mean of 4.5 bowel movements/day). All patients (100.0%) had bloody diarrhea and abdominal pain, while 83.3% and 66.6% had anorexia and weight loss, respectively. In *C. difficile*-infected patients, ALL was diagnosed in 66.6% and NHL in 25.0%. According to the season, the highest positive rate was related to autumn (6 cases-50.0%), followed by winter and summer (3 cases each-25.0%), with a significant relationship between autumn and infection. In one case (0.76%), co-infection of *Aeromonas* spp. and toxigenic *C. difficile* (*tcdA*^*+*^) was detected.

Among our patients, 2 cases (1.5%) were positive for *C. jejuni* with the m ± SD age of 11.0 ± 0.0 months, and *C. coli* was not detected in any case. Both patients were infected in autumn, and were female and ALL^+^. All patients had bloody diarrhea with an average duration of 1.50 ± 0.70 days (mean of 6.5 bowel movements/day). In terms of clinical symptoms, fever, anorexia, and weight loss were seen in all of the cases, while cramp and abdominal pain were not observed in any of them. Interestingly, both *C. jejuni*-positive patients had a co-infection, one with non-toxigenic *C. difficile* and the other with *Aeromonas* spp.

## Discussion

Bacterial gastroenteritis is one of the most important causes of health problems and even death of the elderly and children in different countries [35, 36]. Infectious diarrhea is very important in patients with special immune states in all age groups, especially those who are immunocompromised [37–39]. Of these, bacteria account for a significant number of infections and are considered the main cause of diarrheal disease [8, 15]. Few studies have been reported in the world about the incidence of diarrhea caused by *Aeromonas* spp., *C. jejuni*, *C. coli*, and toxigenic *C. difficile* among patients with underlying disorders. Part of the underreporting is due to the lack of isolation of these bacteria. Various methods are used to identify infectious agents, from conventional methods to rapid tests [19, 40, 41] and due to the special growth conditions of our target bacteria and the lack of molecular facilities in most clinical laboratories, the infection caused by them is not well diagnosed. Therefore, our aim was to simultaneously investigate the infection caused by them among immunocompromised hosts with diarrhea for the first time in Iran.

Our results showed that 14.6% of children with blood malignancies were positive for *Aeromonas* spp. infection, which was similar to other studies conducted among patients with special immune conditions. In a study by Obi et al., in Limpopo, South Africa, *Aeromonas* species were identified in 13.3% of HIV-infected patients with chronic diarrhea in a rural area [42]. In another study in Spain, patients with various diseases, including hematological malignancies, cirrhosis of the liver, hospitalized in ICU, organ transplants, and HIV^+^, were identified with a variety of *Aeromonas* infections, most of them had *Aeromonas* gastroenteritis [43]. Also, reports of rare species of *Aeromonas* in patients with special conditions are interesting. For example, Ana Fernández-Bravo et al. identified *Aeromonas trota* in the diarrhea sample of a 69-year-old Spanish woman with a history of ovarian cancer [15].

Although there has been no report on the prevalence of diarrhea caused by *Aeromonas* spp. in children with cancer in Iran, only a few researches in this field have been conducted on this age group with normal immune system. In some studies, the prevalence of *Aeromonas* spp. was 3.1% in Tehran [44], 0.5% in Zanjan and Qazvin [45], and 1.0% in Arak [46]. In the study done by Rahimi-Larki et al. on samples of children with diarrhea in India (2006), only 1.0% of the patients were positive for *A. hydrophila* [47]. In a study conducted in some Asian countries and several locations in Africa, the relationship between *Aeromonas* and the incidence of moderate-to-severe diarrhea (MSD) in children under 5 years of age was investigated, and their results showed that 22.2% of cases were positive [48].

According to the season, the highest positive rate was related to the autumn with 13 cases (68.0%), followed by winter with 4 cases (21.0%). This result is similar to the results obtained by Ghenghesh et al. in Libya, where the highest positive rate of *Aeromonas* was determined in autumn with 20.8% and in winter with 11.9% [49].

In our study, 9.2% of diarrheal samples were positive for *C. difficile*, and only in one case, the *tcdA* toxin gene was detected. A study by Zargar et al. in Qom, which surveyed *C. difficile* infection in adults with diarrhea, showed that 11 out of 68 patients were positive for this bacterium [50]. Other studies have been carried out in the presence of *C. difficile* in the feces of hospitalized patients in the world as well as in Iran. In Hematyar’s study in northeastern Iran, 4.7% of stool samples from patients hospitalized in several hospitals were positive for *C. difficile* [51]. In the study by Armin et al. in Tehran, *C. difficile* was detected in 45.2% of hospitalized children with diarrhea [52]. In a study in Kenya, 37.6% of children with diarrhea under 5 years of age [53], and in Brazil, 5.5% of children with acute diarrhea were positive for this bacterium [54].

Reports of *C. difficile* infections in immunocompromised children are rare worldwide. In a study conducted in Tehran (2008–2009), out of 152 children with underlying conditions, 25% were positive for *C. difficile* by culture, and 92% of these isolates harbored toxins A and B [55]. In another study in Tehran (2011–2012), *C. difficile* was detected in 17.14% of stool samples of children with cancer by multiplex PCR method, and 72% of the isolates were toxin-producing strains. Among their strains, A^−^/B^+^ (8 cases) were detected more than A^+^/B^−^ (4 cases) [56]. Price and colleagues in Canada investigated the incidence and characteristics of CDI in children with acute myeloid leukemia (AML). CDI occurred in only 11.0% of children undergoing chemotherapy for AML, suggesting that the prevalence of CDI and its associated outcomes is not an important issue in children with AML [57].

In our study, the prevalence of *C. jejuni* was 1.5%, where both children were 11 months old and had ALL. Several studies have been conducted to investigate the prevalence of *Campylobacter* in children with a normal immune system in Iran and the world. Ghorbanalizadgan et al. in Iran found the prevalence of *Campylobacter* in children under 5 years to be 4%, of which 94% were *C*. *jejuni* and 6% were *C. coli* [58]. Terefe et al. in Ethiopia detected the prevalence of *Campylobacter* in children under 2 years old by PCR method to be 50% [59]. In Ecuador, the prevalence of this bacterium in healthy children was 13.4% [60]. Samie et al. in South Africa showed the prevalence of *Campylobacter* in children with diarrhea (13.2%) and without diarrhea (12.4%) [61].

The prevalence of *Campylobacter* spp. in children with the underlying disease has been investigated in limited studies worldwide. In a study in Pennsylvania, zoonotic infections in children with acute leukemia were investigated, and *Campylobacter* enteritis was one of the causes of infection among the patients [62]. Some other studies have presented reports of extraintestinal cases of *Campylobacter* species. For example, Anvarinejad in Iran identified bacteremia caused by *C. jejuni* in a 14-year-old child with ALL who was undergoing chemotherapy [63]. In a case report in Italy, *C. jejuni* was detected in the blood of two 4- and 7-year-old children with ALL [64]. Recurrent *C. jejuni* bacteremia was also reported in an 18-year-old patient with hypogammaglobulinemia in South Korea [65].

Infectious diseases are usually caused by one type of pathogen. In some cases, infections with two or more pathogens is observed [66]. Co-infections for the outcome of a disease, depending on the level of interactions such as fluctuating host immune response, and drug/diagnostic interventions can be detrimental, insignificant, or beneficial [67]. In our study, co-infections of *C. jejuni* with non-toxigenic *C. difficile* and *Aeromonas* spp.; and *Aeromonas* spp. with toxigenic *C. difficile* were observed in three patients. It seems that the cause of this co-infection can be related to the immunocompromised states of our patients due to cancer and as well as chemotherapy. These co-infections in children suffering from cancer and with gastrointestinal symptoms have not been discussed either in the world. However, co-infections from these bacteria have been reported in hosts with a normal immune system. Barati et al. in Iran reported that 3.5% of *Campylobacter*-positive children were co-infected with intestinal parasites [68]. De Graaf et al. reviewed co-infections in children with diarrhea who tested positive for *C. difficile*. In their study, the percentage of co-infections reported in *C. difficile*-positive children was 20.7% [69]. In addition, Vasilev and colleagues in Bulgaria identified three intestinal pathogens, including *A. hydrophila*, *C. difficile*, and rotavirus in the feces of a 41-year-old female patient who had recurrent diarrhea and abdominal pain [70].

## Conclusion

This is the first study that investigates the simultaneous presence of a number of important bacteria, including *Aeromonas* spp., *C. jejuni*, *C. coli*, and toxigenic *C. difficile*, in children with immunocompromised states who have diarrhea. Although there was no significant relationship between the type of infection and other variables, our results showed that these bacteria have a relatively low prevalence in our region and their presence is important and can cause gastroenteritis in children, especially during underlying diseases. Co-infections with these bacteria were also significant for us. It seems that clinical laboratories should pay more attention to the isolation and identification of these bacteria. Improving the level of hygiene and increasing monitoring of food and water can be effective in controlling diarrheal diseases caused by bacteria. In addition, it is recommended to investigate the origin of these bacteria along with the evaluation of their virulence factors in future studies. Also, most of the patients were undergoing chemotherapy during this study, and the presence of bloody or watery diarrhea may be due to the side effects of this therapeutic procedure. This is a confounding variable and needs further investigation in further research. In addition, viral agents or other pathogens could also have caused the disease in our patients, which is under investigation in our future project. Since asymptomatic colonization with some bacteria is common, it is recommended to perform quantitative-PCR to distinguish infection from colonization in subsequent studies.

### Limitations of the study

One of our limitations was not using conventional methods to isolate target bacteria due to their fastidious nature and time constraints. Investigation of other bacterial agents causing diarrhea was not evaluated at this phase due to financial constraints. Another limitation was not adding a control group with normal immune states associated with infectious diarrhea and/or with immunocompromised states associated with non-infectious diarrhea. Therefore, diarrhea may be caused by side effects of chemotherapy rather than pathogens [10], which could not be investigated in this study. In addition, since the clinical spectrum caused by *C. difficile* varies from asymptomatic colonization to sepsis and death [71], unfortunately, in this study, it was not possible to investigate the colonization from infection for non-toxigenic *C. difficile* with PCR alone.

## Data Availability

The datasets used and/or analyzed during the current study are available from the corresponding author on reasonable request.

## Abbreviations

CDIs: *C. difficile* infections
ALL: Acute lymphocytic leukemia
NHL: Non-hodgkin’s lymphoma
PCR: Polymerase chain reaction
ATCC: American type culture collection
*tcd*: Toxigenic *C. difficile*
*cadF*: *Campylobacter* adhesion to fibronectin
*cdt*: *C. difficile* binary toxin
16S rRNA: 16 S ribosomal RNA
*cdd3*: *C. difficile* downstream 3
